# A prospective observational study of early intervention with erythropoietin therapy and renal survival in non-dialysis chronic kidney disease patients with anemia: JET-STREAM Study

**DOI:** 10.1007/s10157-015-1225-9

**Published:** 2016-01-16

**Authors:** Tadao Akizawa, Yoshiharu Tsubakihara, Hideki Hirakata, Yuzo Watanabe, Hiroki Hase, Shinichi Nishi, Tetsuya Babazono, Michiko Kumagai, Shingo Katakura, Yukari Uemura, Yasuo Ohashi, JET-STREAM Study Group

**Affiliations:** 1 Division of Nephrology, Department of Medicine, Showa University School of Medicine, 1-5-8 Hatanodai, Shinagawa-ku, Tokyo, 142-8666 Japan; 2 Course of Safety Management in Health Care Sciences, Graduate School of Health Care Sciences, Jikei Institute, Osaka, Japan; 3 Division of Nephrology and Dialysis Center, Japanese Red Cross Fukuoka Hospital, Fukuoka, Japan; 4 Department of Internal Medicine, Kasugai Municipal Hospital, Nagoya, Japan; 5 Department of Nephrology, Toho University Ohashi Medical Center, Tokyo, Japan; 6 Division of Nephrology and Kidney Center, Kobe University Graduate School of Medicine, Kobe, Japan; 7 Division of Nephrology and Hypertension, Diabetes Center, Tokyo Women’s Medical University School of Medicine, Tokyo, Japan; 8 Pharmacovigilance Department, Chugai Pharmaceutical Co. Ltd., 2-1-1 Nihonbashi-Muromachi, Chuo-ku, Tokyo, 103-8324 Japan; 9 Biostatistics Division, Clinical Research Support Center, The University of Tokyo Hospital, Tokyo, Japan; 10 Department of Integrated Science and Engineering for Sustainable Society, Chuo University, Tokyo, Japan

**Keywords:** Anemia, Chronic kidney disease, Erythropoiesis-stimulating agents, Non-dialysis, Renal survival

## Abstract

**Background:**

There is limited data showing that early treatment for anemia could prolong renal survival in non-dialysis chronic kidney disease (CKD) patients. We therefore investigated the relationship between hemoglobin (Hb) levels at initiation of epoetin beta therapy and renal outcome in non-dialysis CKD patients with anemia.

**Methods:**

In this prospective, multi-center, observational study, non-dialysis CKD patients with anemia who were naïve to erythropoiesis-stimulating agents (ESAs) were divided into three groups based on their Hb levels at initiation of epoetin beta therapy (Group I: 10 ≤ Hb < 11 g/dL, Group II: 9 ≤ Hb < 10 g/dL, and Group III: Hb < 9 g/dL). The primary endpoint was time to first occurrence of any renal event. For the primary analysis, an inverse probability weighted Cox regression model was used to adjust time-dependent selection bias in the artificially censored data.

**Results:**

A total of 1113 patients were eligible for primary endpoint analysis. Risk of renal events was significantly higher in Group III compared with Group I (HR, 2.52; 95 % CI, 1.98–3.21; *P* < 0.0001); although not significant, the risk was also higher in Group II compared with Group I (HR, 1.48; 95 % CI, 0.91–2.40; *P* = 0.11).

**Conclusion:**

Initiation of ESA therapy when Hb levels decreased below 11 g/dL but not below 10 g/dL could be more effective at reducing the risk of renal events in non-dialysis CKD patients with anemia compared with initiation of ESA therapy at below 9 g/dL or even 10 g/dL.

## Introduction

Anemia is a common complication in patients with chronic kidney disease (CKD) and is primarily caused by declining erythropoietin production in such patients [1]. Anemia can worsen renal and cardiac function and is associated with an increased risk of mortality or hospitalization [2]. Thus, treatment of anemia in CKD patients is especially important. Erythropoiesis-stimulating agents (ESAs) have been used for the treatment of anemia in such patients. Correction of anemia with ESAs is associated with improved outcome [3] and quality of life [4].

There have been many reports of ESA studies, and some have discussed appropriate target hemoglobin (Hb) levels for maintenance with ESAs. The CHOIR study failed to show the benefit of setting a high target Hb level and suggested a potential for increased composite risk of death and cardiovascular events [5]. The TREAT study also showed that setting a high target Hb level provided no clinical benefit and, instead, increased the risk of cerebrovascular disease [6]. The appropriate target Hb level for ESA therapy remains controversial.

Still, there have been few reports on an appropriate Hb level for starting ESA therapy. Although the 2012 guideline from Kidney Disease: Improving Global Outcomes states that introduction of ESA therapy should be considered when Hb level decreases below 10 g/dL in patients with non-dialysis-dependent CKD [7], it provides no evidence to support this recommendation. Some evidence is provided by Gouva et al., who found that early intervention with ESAs in anemia slows the progression of renal disease and delays the initiation of renal replacement therapy [8]. Although early detection and management of anemia is considered to be vital, the best timing for starting ESA therapy is still uncertain, and it is now imperative that we collect data on the appropriate Hb level for starting ESA therapy.

The JET-STREAM (Japan Erythropoietin Treatment survey for STarting hemoglobin level in REnal Anemia Management) study was conducted to investigate the relationship between renal outcome and the Hb level at initiation of epoetin beta therapy, rather than the target Hb level, in non-dialysis CKD patients with anemia.

## Methods

### Study population

Patients were recruited from February 2010 to March 2011. Eligible patients were non-dialysis, ESA-naïve, CKD patients with anemia who were scheduled to start epoetin beta therapy. Patients were not scheduled for renal replacement therapy within at least the following 6 months. Patients with non-renal anemia or with an estimated glomerular filtration rate (eGFR) [9] of less than 6 mL/min/1.73 m^2^ were excluded. All study participants provided written informed consent.

### Study design and measurements

This study was a prospective, observational study. Epoetin beta (EPOGIN 1500, 3000, 6000, 9000 and 12,000 IU, Chugai Pharmaceutical, Tokyo, Japan) was used for this study according to the package insert approved by the Ministry of Health, Labour and Welfare in Japan [10]. Patients were followed up for a maximum of 2 years from initiation of epoetin beta therapy or until discontinuation of therapy, initiation of renal replacement therapy, death, malignancy, withdrawal of consent, or loss of follow-up.

Information was collected on patient baseline characteristics (age, sex, medical history, comorbidities), epoetin beta treatment status, treatment status of any other drugs, inpatient/outpatient status, the date renal replacement therapy was introduced, laboratory test values, and adverse reactions. Data on Hb and serum creatinine (sCr) levels were collected not only during the epoetin beta treatment period but also retrospectively on the dates that Hb levels decreased below 11 g/dL for the first time.

The study protocol was approved by each local institutional review board (approval no. at Osaka General Medical Center: 21-562), and the study was conducted in accordance with the Declaration of Helsinki and Japanese Ministry of Health, Labour and Welfare regulations for postmarketing surveillance. This study is registered with the University Hospital Medical Information Network (ID: UMIN000003116).

The primary endpoint was time to first occurrence of any renal event (defined as initiation of renal replacement therapy, doubling of sCr level, or measurement of eGFR less than 6.0 mL/min/1.73 m^2^). Secondary endpoints were occurrence of cardiovascular events (defined as death or hospitalization from heart failure, angina, myocardial infarction, cerebral infarction, intracranial cerebral hemorrhage, or transient ischemic attack) and safety. Relationships between patient baseline characteristics and outcomes were also evaluated.

### Statistical analysis

**Fig. 1:**
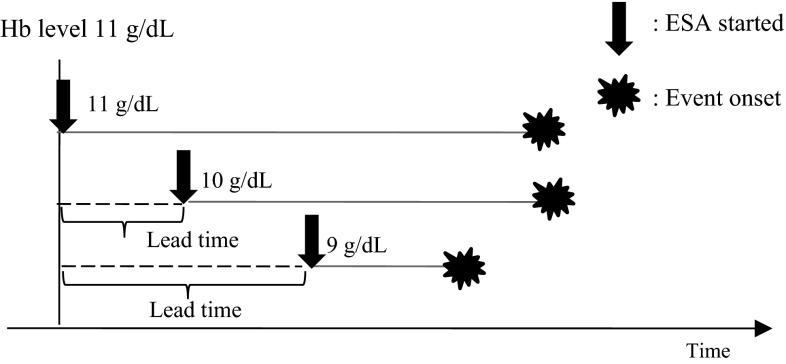
Study design accounting for lead-time bias. To account for lead-time bias, the date, Hb levels, and sCr levels were confirmed at the time Hb levels decreased below 11 g/dL for the first time. Analysis was started from the point Hb levels decreased below 11 g/dL

Eligible patients were divided into the following three groups based on their Hb levels at initiation of epoetin beta therapy: Group I consisted of patients with 10 ≤ Hb < 11 g/dL, Group II with 9 ≤ Hb < 10 g/dL, and Group III with Hb < 9 g/dL. If the time of epoetin beta therapy initiation was defined as the starting point of survival analysis, later starters may be at a disadvantage because of the lead time from the earlier stage to therapy initiation (i.e., their Hb levels may be higher) and because of their relatively poor physical condition compared with earlier starters. To account for this lead-time bias, the primary endpoint was analyzed from the day Hb levels decreased below 11 g/dL for the first time (Fig. 1), and a recently developed statistical concept called “dynamic treatment regime” [11] was applied for group comparisons.

For comparison of the three groups, we analyzed Group I vs Group II and Group I vs Group III. The treatment strategy for each group was dynamic in that the decision to initiate treatment was guided by each patient’s developing clinical status (in this study, Hb level). Hernan et al. [11] have shown that the relative efficacy of a dynamic treatment regime can be evaluated and compared using inverse probability weighting (IPW), which was proposed by Robins et al. [12]. To compare two dynamic treatment regimes, we artificially censored those patients who deviated from one of the two regimes of interest; however, uncensored patients may have different risk factor profiles from censored patients. We used the IPW Cox regression model to adjust for this time-dependent selection bias in the artificially censored patients. We estimated each patient-specific weight using the inverse of each patient’s estimated probability of remaining uncensored. These probabilities were estimated by fitting a pooled logistic regression model to the conditional probability of the remaining uncensored group at each visit given the history of covariates. The covariates in the model included baseline- and time-dependent risk factors for renal and cardiovascular events: Hb levels, sCr levels, age, sex, and comorbidities observed in more than 5 % of patients in each group (i.e., hypertension, heart failure, angina, arrhythmia, diabetes, hyperlipidemia, and hyperuricemia). To increase precision in estimation, we used a stabilized weight with the numerator representing the probability of each patient remaining uncensored given only the baseline covariates and the denominator with time-dependent covariates [13]. Data missing from continuous baseline variables were substituted by the baseline mean value of all patients.

The Cox regression model was used to assess relationships between patient baseline characteristics (including Hb levels, sCr levels, age, sex, comorbidities, and medical history; comorbidities and medical histories observed in more than 5 % of patients in each group and with less than 20 % of data missing were included) and outcomes for those patients eligible for efficacy analysis. Values of *P* < 0.05 were considered statistically significant. All analyses were conducted using SAS v9.3 (SAS Institute, Cary, NC, USA).

## Results

### Patients and baseline characteristics

**Fig. 2:**
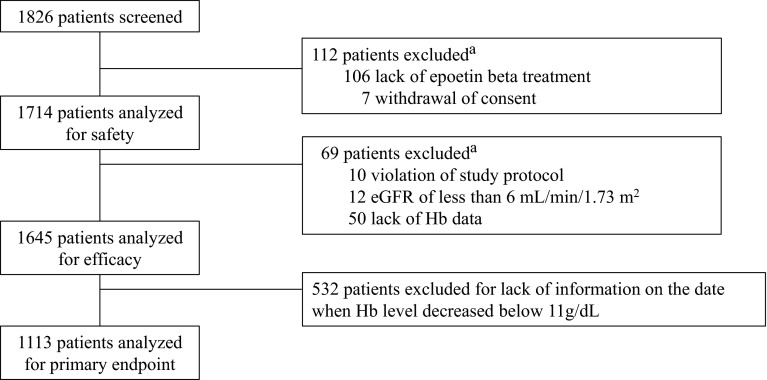
Study profile. Of 1826 patients screened, 112 were excluded because they did not receive epoetin beta treatment or they withdrew consent. The remaining 1714 patients were eligible for safety analysis in this study. 1645 patients were eligible for efficacy analysis, and 1113 patients with information on the date when Hb levels decreased below 11 g/dL were eligible for analysis of the primary endpoint. ^a^Numbers of excluded patients in this figure overlap

Of 1826 patients screened, 112 patients were excluded because they withdrew consent or did not receive epoetin beta therapy, leaving 1714 patients eligible for safety analysis in this study. A total of 1645 patients were eligible for efficacy analysis, and 1113 patients were eligible for analysis of the primary endpoint (Fig. 2). Patients who were eligible for primary endpoint analysis were divided into 3 groups based on Hb levels at initiation of epoetin beta: Group I (10 ≤ Hb < 11 g/dL) had 309 patients, Group II (9 ≤ Hb < 10 g/dL) had 545 patients, and Group III (Hb < 9 g/dL) had 259 patients. The characteristics of these patients are summarized in Table 1. The mean eGFR levels on the day the Hb levels decreased below 11 g/dL for the first time in Groups I, II, and III were 23.6 ± 12.3 mL/min/1.73 m^2^ (median, 20.7 mL/min/1.73 m^2^), 24.6 ± 13.1 mL/min/1.73 m^2^ (median, 21.8 mL/min/1.73 m^2^), and 27.7 ± 16.8 mL/min/1.73 m^2^ (median, 22.9 mL/min/1.73 m^2^), respectively. At this time, the respective Hb levels in Groups I, II, and III were 10.5 ± 0.4 g/dL, 10.5 ± 0.5 g/dL, and 10.4 ± 0.5 g/dL (Table 2).

**Table 1:** Characteristics of patients

Variables	Patients analyzed for efficacy (*N* = 1645)	Patients analyzed for primary endpoint (*N* = 1113)	Sub-groups based on Hb levels at initiation of epoetin beta			*P* value
			Group I	Group II	Group III	
			10 ≤ Hb < 11 g/dL (*N* = 309)	9 ≤ Hb < 10 g/dL (*N* = 545)	Hb < 9 g/dL (*N* = 259)	
Sex						
Male	1039 (63.2 %)	727 (65.3 %)	222 (71.8 %)	348 (63.9 %)	157 (60.6 %)	0.012
Age (years)^a^	70.5 ± 12.6	70.5 ± 12.4	69.0 ± 11.9	70.7 ± 12.8	71.9 ± 12.1	0.021
Cause of CKD						
Chronic glomerulonephritis	345 (21.0 %)	249 (22.4 %)	64 (20.7 %)	130 (23.9 %)	55 (21.2 %)	0.50
Diabetic nephropathy	579 (35.2 %)	388 (34.9 %)	113 (36.6 %)	179 (32.8 %)	96 (37.1 %)	0.38
Nephrosclerosis	391 (23.8 %)	268 (24.1 %)	80 (25.9 %)	129 (23.7 %)	59 (22.8 %)	0.66
Medical history						
Heart failure	91 (5.5 %)	58 (5.2 %)	18 (5.8 %)	25 (4.6 %)	15 (5.8 %)	0.66
Angina	92 (5.6 %)	67 (6.0 %)	19 (6.1 %)	30 (5.5 %)	18 (6.9 %)	0.72
Arrhythmia	54 (3.3 %)	42 (3.8 %)	9 (2.9 %)	15 (2.8 %)	18 (6.9 %)	0.0091
Myocardial infarction	83 (5.0 %)	55 (4.9 %)	12 (3.9 %)	26 (4.8 %)	17 (6.6 %)	0.33
Peripheral arterial disease	20 (1.2 %)	15 (1.3 %)	4 (1.3 %)	7 (1.3 %)	4 (1.5 %)	0.95
Cerebral infarction	181 (11.0 %)	130 (11.7 %)	36 (11.7 %)	63 (11.6 %)	31 (12.0 %)	0.99
Cerebral hemorrhage	39 (2.4 %)	28 (2.5 %)	7 (2.3 %)	14 (2.6 %)	7 (2.7 %)	0.94
Malignancy	216 (13.1 %)	149 (13.4 %)	41 (13.3 %)	69 (12.7 %)	39 (15.1 %)	0.65
Complications						
Hypertension	1353 (82.2 %)	919 (82.6 %)	246 (79.6 %)	462 (84.8 %)	211 (81.5 %)	0.14
Heart failure	149 (9.1 %)	87 (7.8 %)	24 (7.8 %)	32 (5.9 %)	31 (12.0 %)	0.011
Cardiac hypertrophy	56 (3.4 %)	30 (2.7 %)	9 (2.9 %)	15 (2.8 %)	6 (2.3 %)	0.90
Angina	129 (7.8 %)	94 (8.4 %)	23 (7.4 %)	49 (9.0 %)	22 (8.5 %)	0.74
Arrhythmia	100 (6.1 %)	69 (6.2 %)	18 (5.8 %)	34 (6.2 %)	17 (6.6 %)	0.93
Myocardial infarction	38 (2.3 %)	26 (2.3 %)	8 (2.6 %)	12 (2.2 %)	6 (2.3 %)	0.94
Peripheral arterial disease	48 (2.9 %)	34 (3.1 %)	14 (4.5 %)	13 (2.4 %)	7 (2.7 %)	0.20
Cerebral infarction	73 (4.4 %)	46 (4.1 %)	16 (5.2 %)	16 (2.9 %)	14 (5.4 %)	0.14
Cerebral hemorrhage	3 (0.2 %)	3 (0.3 %)	1 (0.3 %)	2 (0.4 %)	0 (0.0 %)	0.81
Transient ischemic attack	7 (0.4 %)	6 (0.5 %)	2 (0.6 %)	2 (0.4 %)	2 (0.8 %)	0.77
Diabetes mellitus	734 (44.6 %)	495(44.5 %)	145 (46.9 %)	228 (41.8 %)	122 (47.1 %)	0.22
Diabetic retinopathy	244 (14.8 %)	167 (15.0 %)	43 (13.9 %)	81 (14.9 %)	43 (16.6 %)	0.67
Hyperlipidemia	499 (30.3 %)	369 (33.2 %)	111 (35.9 %)	185 (33.9 %)	73 (28.2 %)	0.13
Hepatitis B	11 (0.7 %)	7 (0.6 %)	2 (0.6 %)	4 (0.7 %)	1 (0.4 %)	0.90
Hepatitis C	56 (3.4 %)	39 (3.5 %)	10 (3.2 %)	21 (3.9 %)	8 (3.1 %)	0.82
Gastrointestinal ulcer	62 (3.8 %)	36 (3.2 %)	10 (3.2 %)	13 (2.4 %)	13 (5.0 %)	0.14
Secondary hyperparathyroidism	62 (3.8 %)	42 (3.8 %)	11 (3.6 %)	22 (4.0 %)	9 (3.5 %)	0.90
Hyperphosphatemia	47 (2.9 %)	29 (2.6 %)	4 (1.3 %)	15 (2.8 %)	10 (3.9 %)	0.15
Hyperuricemia	503 (30.6 %)	363 (32.6 %)	96 (31.1 %)	176 (32.3 %)	91 (35.1 %)	0.57
History of smoking	445 (27.1 %)	313 (28.1 %)	87 (28.2 %)	155 (28.4 %)	71 (27.4 %)	0.96
History of hospitalization	911 (55.4 %)	665 (59.7 %)	161 (52.1 %)	335 (61.5 %)	169 (65.3 %)	0.033
History of blood transfusion	103 (6.3 %)	68 (6.1 %)	11 (3.6 %)	36 (6.6 %)	21 (8.1 %)	0.097
Systolic blood pressure (mmHg)^a^	136.3 ± 20.0 (*N* = 1331)	136.2 ± 19.7 (*N* = 924)	138.1 ± 19.6 (*N* = 262)	134.5 ± 19.5 (*N* = 445)	137.2 ± 20.1 (*N* = 217)	0.045
Diastolic blood pressure (mmHg)^a^	72.1 ± 13.0 (*N* = 1325)	72.1 ± 13.0 (*N* = 920)	75.1 ± 13.6 (*N* = 262)	71.2 ± 12.3 (*N* = 443)	70.1 ± 13.0 (*N* = 215)	<0.0001
Total protein (g/dL)^a^	6.6 ± 0.7 (*N* = 1354)	6.6 ± 0.7 (*N* = 914)	6.8 ± 0.6 (*N* = 255)	6.6 ± 0.8 (*N* = 453)	6.4 ± 0.8 (*N* = 206)	<0.0001
Ferritin (ng/mL)^a^	185.1 ± 183.4 (*N* = 710)	176.3 ± 181.0 (*N* = 457)	160.1 ± 139.3 (*N* = 137)	165.7 ± 140.6 (*N* = 220)	222.0 ± 279.0 (*N* = 100)	0.016
TSAT ( %)^a^	27.8 ± 11.5 (*N* = 680)	28.6 ± 10.6 (*N* = 436)	29.9 ± 10.7 (*N* = 138)	28.9 ± 10.7 (*N* = 203)	26.3 ± 10.2 (*N* = 95)	0.037
Albumin (g/dL)^a^	3.6 ± 0.6 (*N* = 1431)	3.6 ± 0.6 (*N* = 973)	3.8 ± 0.5 (*N* = 268)	3.7 ± 0.5 (*N* = 480)	3.4 ± 0.6 (*N* = 225)	<0.0001
Calcium (mg/dL)^a^	8.7 ± 0.7 (*N* = 1341)	8.7 ± 0.7 (*N* = 908)	8.9 ± 0.6 (*N* = 250)	8.7 ± 0.6 (*N* = 445)	8.5 ± 0.7 (*N* = 213)	<0.0001
Phosphorus (mg/dL)^a^	3.9 ± 0.8 (*N* = 1237)	3.9 ± 0.8 (*N* = 833)	3.8 ± 0.8 (*N* = 222)	3.8 ± 0.7 (*N* = 409)	4.1 ± 0.9 (*N* = 202)	0.0001
Total cholesterol (mg/dL)^a^	175.7 ± 40.9 (*N* = 1056)	174.6 ± 38.9 (*N* = 704)	178.0 ± 38.0 (*N* = 201)	175.1 ± 40.4 (*N* = 341)	169.5 ± 36.4 (*N* = 162)	0.11
C-reactive protein (mg/dL)^a^	0.5 ± 1.5 (*N* = 915)	0.5 ± 1.6 (*N* = 583)	0.4 ± 1.1 (*N* = 139)	0.3 ± 0.6 (*N* = 287)	1.0 ± 2.7 (*N* = 157)	<0.0001
Urine protein/creatinine^a^	2.8 ± 5.2 (*N* = 716)	2.9 ± 5.9 (*N* = 480)	3.2 ± 9.0 (*N* = 133)	2.4 ± 3.9 (*N* = 240)	3.5 ± 4.6 (*N* = 107)	0.22
Starting dose of epoetin beta (IU/month)^a^	12806.4 ± 6792.4 (*N* = 1436)	12135.8 ± 6757.1 (*N* = 972)	11123.1 ± 6085.6 (*N* = 260)	11758.0 ± 6608.9 (*N* = 471)	13966.8 ± 7380.1 (*N* = 241)	<0.0001

*N* number of patients, *SD* standard deviation, *CKD* chronic kidney disease, *TSAT* transferrin saturation

^a^Values were mean ± SD

**Table 2:** Lead time and changes of laboratory test values

Variables	Group I	Group II	Group III	*P* value
	10 ≤ Hb < 11 g/dL (*N* = 309)	9 ≤ Hb < 10 g/dL (*N* = 545)	Hb < 9 g/dL (*N* = 259)	
When Hb levels decreased below 11 g/dL for the first time				
Hb (g/dL)^a^	10.5 ± 0.4	10.5 ± 0.5	10.4 ± 0.5	0.027
sCr (mg/dL)^a^	2.7 ± 1.3	2.4 ± 1.2	2.3 ± 1.2	0.0009
eGFR (mL/min/1.73 m^2^)^a^	23.6 ± 12.3	24.6 ± 13.1	27.7 ± 16.8	0.0014
When epoetin beta therapy was initiated				
Hb (g/dL)^a^	10.4 ± 0.3	9.5 ± 0.3	8.3 ± 0.6	<0.0001
sCr (mg/dL)^a^	2.9 ± 1.4	3.0 ± 1.3	3.4 ± 1.5	<0.0001
eGFR (mL/min/1.73 m^2^)^a^	21.4 ± 11.0	18.5 ± 8.7	16.8 ± 8.1	<0.0001

*Hb* hemoglobin, *sCr* serum creatinine, *eGFR* estimated glomerular filtration rate

^a^Values were mean ± SD

### Primary endpoint

Renal events occurred in 100 patients (32.4 %) in Group I, 246 patients (45.1 %) in Group II, and 157 patients (60.6 %) in Group III.

The calculated weights between Groups I and II and between Groups I and III are summarized in Table 3. When Groups I and II were compared, some patients had extreme weights. It is well known that such extreme weights result in a large mean square of the estimated effect. To account for these extreme weights, we truncated the weight at the 99th percentile. That is, weights larger than the 99th percentile were set to the value of the 99th percentile. When Groups I and III were compared, the calculated weights were not as large, and truncation of the weights was unnecessary. After adjusting for artificial censoring using the IPW method, the risk of renal events in Group III was significantly higher than in Group I (HR, 2.52; 95 % CI, 1.98–3.21; *P* < 0.0001); and although not significant, this risk was higher in Group II than in Group I (HR, 1.48; 95 % CI, 0.91–2.40; *P* = 0.11) (Table 3A). For sensitivity analysis, we also used a weight truncated at the 98th percentile to compare Groups I and II. Risk of renal events in Group II was significantly higher than in Group I (HR, 1.29; 95 % CI, 1.02–1.64; *P* = 0.033) (Table 3B).

**Table 3:** Adjusted hazard ratio for renal events

	Hazard ratio	95 % CI	*P* value
(A) Hazard ratio after adjusting for data of patients with weight above 99th percentile in Group II			
Group I	Reference		
Group II	1.48	0.91–2.40	0.11
Group III	2.52	1.98–3.21	<0.0001
(B) Hazard ratio after adjusting for data of patients with weight above 98th percentile in Group II			
Group I	Reference		
Group II	1.29	1.02–1.64	0.033

For weight calculation, the time-dependent covariates included in the model are sCr levels and Hb levels. We also included the following baseline covariates: age, sex, hypertension, heart failure, angina, arrhythmia, diabetes, hyperlipidemia, and hyperuricemia

In the comparison between Groups I and II, there were subjects with extreme weights (median 0.84, 98th percentile 3.74, 99th percentile 34.23, maximum 236,242.07). To account for these extreme weights, we compared the groups using weights truncated at the 99th percentile and 98th percentile

*CI* confidence interval

### Secondary endpoints

Cardiovascular events occurred in 24 patients (7.8 %) in Group I, 52 patients (9.5 %) in Group II, and 31 patients (12.0 %) in Group III. Since the weights were extremely large for the comparison between Groups I and II, we truncated the weights at the 98th percentile. Based on the IPW method, the risk of cardiovascular events did not differ between Group I and Group II, and this risk in Group III was higher than in Group I, but not significantly (HR, 1.94; 95 % CI, 0.96–3.94; *P* = 0.066) (Table 4).

**Table 4:** Adjusted hazard ratio for cardiovascular events

	Hazard ratio	95 % CI	*P* value
Hazard ratio after adjusting for data of patients with weight above 98th percentile in Group II			
Group I	Reference		
Group II	1.00	0.58–1.71	0.99
Group III	1.94	0.96–3.94	0.066

For weight calculation, the time-dependent covariates included in the model are sCr levels and Hb levels. We also included the following baseline covariates: age, sex, hypertension, heart failure, angina, arrhythmia, diabetes, hyperlipidemia and hyperuricemia. In the comparison between Groups I and II, there were subjects with extreme weights (median 1.03, 98th percentile 6.18, 99th percentile 96.29, maximum 495,481.07). To account for these extreme weights, we compared the groups using weights truncated at the 98th percentile

*CI* confidence interval

**Fig. 3:**
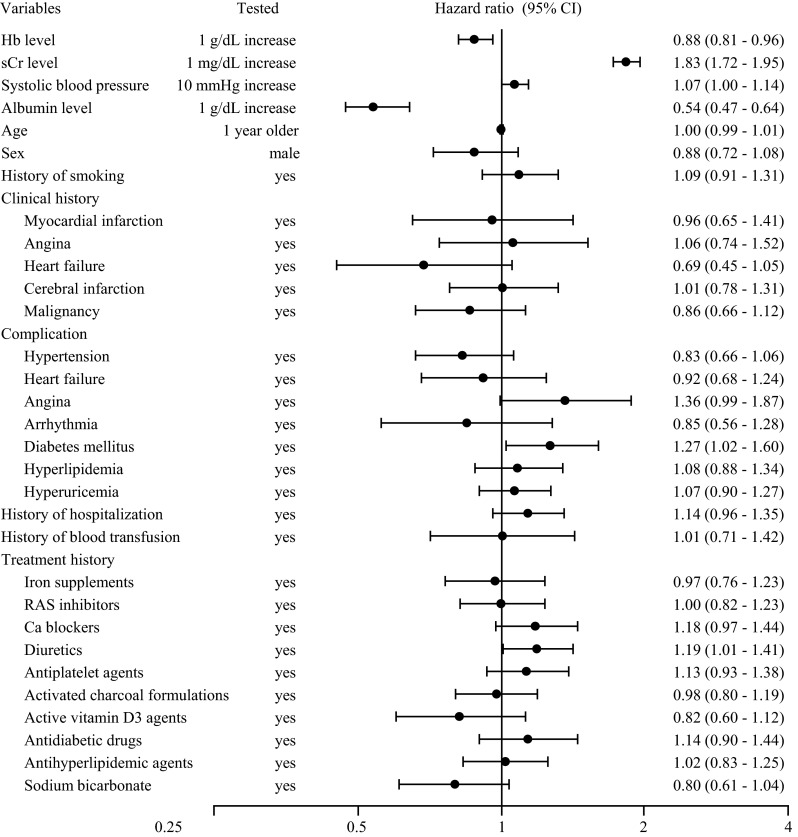
Prognosis factors for renal survival. *Hb* hemoglobin, *sCr* serum creatinine, *RAS* renin-angiotensin system, *Ca* calcium, *CI* confidence interval. The Cox regression model was used to assess relationships between patient baseline characteristics (including Hb levels, sCr levels, age, sex, comorbidities, and medical history; comorbidities and medical histories observed in more than 5 % of patients in each group and with less than 20 % of data missing were included) and outcomes for those patients eligible for efficacy analysis

To evaluate the relationships between patient baseline characteristics and outcomes, we used data from 1645 patients. The main statistically significant clinical variables associated with poor renal survival were lower Hb levels, higher sCr levels, lower serum albumin levels, comorbid diabetes mellitus, and previous diuretic use (Fig. 3).

Of the 1714 patients in the safety analysis set, adverse reactions related to epoetin beta were reported in 13 patients (0.8 %) (Table 5). Of these, serious adverse reactions were reported in five patients (0.3 %): cerebral hemorrhage in two patients and cerebral infarction, acute myocardial infarction, and aortic aneurysm rupture in one patient each.

**Table 5:** Adverse reactions (number of patients)

Patients analyzed for safety	Hb levels at initiation of epoetin beta					Total (*N* = 1714)
	11 g/dL ≤ Hb (*N* = 45)	10 ≤ Hb < 11 g/dL (*N* = 352)	9 ≤ Hb < 10 g/dL (*N* = 757)	Hb < 9 g/dL (*N* = 521)	Unknown (*N* = 39)	
Thrombocytopenia		1 (0.3 %)				1 (0.1 %)
Cerebral hemorrhage^a^			2 (0.3 %)			2 (0.1 %)
Cerebral infarction^a^		1 (0.3 %)				1 (0.1 %)
Acute myocardial infarction^a^				1 (0.2 %)		1 (0.1 %)
Aortic aneurysm rupture^a^				1 (0.2 %)		1 (0.1 %)
Hypertension			1 (0.1 %)	1 (0.2 %)		2 (0.1 %)
Pruritus			1 (0.1 %)	1 (0.2 %)		2 (0.1 %)
Chest discomfort			1 (0.1 %)			1 (0.1 %)
Injection site pain		1 (0.3 %)				1 (0.1 %)
Blood pressure increased			1 (0.1 %)			1 (0.1 %)
Total	0 (0.0 %)	3 (0.9 %)	6 (0.8 %)	4 (0.8 %)	0 (0.0 %)	13 (0.8 %)

Incidence of adverse reactions was tabulated for the 1714 patients in the safety analysis set

^a^Serious adverse reactions

## Discussion

A randomized comparative study design would be preferable for exploring the appropriate timing (Hb level) for initiation of ESA therapy. However, such a design presents ethical difficulties given concerns that patients with depleted Hb levels might be deprived of an opportunity for anemia treatment. Therefore, an observational study design becomes the realistic choice, but such a design itself presents two issues that should be noted: lead-time bias and selection bias. And a valid assessment is impossible without first taking these biases into account.

With the first issue, lead-time bias, renal function in a group starting ESA treatment at a lower Hb level will be worse than that in a group starting ESA treatment at a higher Hb level. Therefore, the time to onset of events in the former group will be underestimated because it will appear shorter (Fig. 1). To eliminate such bias in this study, the date, Hb levels, and sCr levels were confirmed at the time Hb levels decreased below 11 g/dL for the first time, and analysis used this data rather than the data from initiation of ESA treatment. Although there was variation in patient background characteristics such as sex, age, and comorbidities, and there was variation, for example, in the eGFR levels of each group when Hb levels decreased below 11 g/dL (Table 2), analysis was performed after using the IPW method to adjust for selection bias, the second issue. As described, analysis was performed in this study on the basis of a study design that resolves the issues that would normally arise in an observational study of the appropriate timing for initiation of ESA therapy.

In analysis of the effects of renal events using the IPW method, to confirm that a comparison of Groups I and II would not change interpretation of the results, both a 99th percentile weight and a 98th percentile weight were used, resulting in the respective hazard ratios 1.48 (95 % CI, 0.91–2.40; *P* = 0.11) and 1.29 (95 % CI, 1.02–1.64; *P* = 0.033). It is known that as weights are progressively truncated, the precision of the estimate increases, resulting in induced bias [13]. Using a 99th percentile weight would provide results closer to the true value but with a wider confidence interval than when using a 98th percentile weight. Generally speaking, even if the hazard ratio is close to 1, a narrow confidence interval could show a significant difference, and although such a difference would be statistically significant, it would have little clinical value (e.g., HR, 1.05; 95 % CI, 1.01–1.09). On the other hand, even without a statistically significant difference, its effect could be sufficiently suggested by a hazard ratio further from 1 if the confidence interval is kept somewhat narrow. The results this time with the 99th percentile weight are similar.

By using the above study design and analysis method, this study demonstrated that initiation of ESA therapy when Hb levels decreased below 11 g/dL could reduce the risk of renal events in non-dialysis CKD patients with anemia more effectively than initiation of ESA therapy at below 9 g/dL. Also, sensitivity analysis showed that initiation of ESA therapy when Hb levels decreased below 11 g/dL could even reduce the risk of renal events more effectively than initiation of ESA therapy at below 10 g/dL.

Starting in 2000, Gouva et al. studied 88 nondiabetic patients in Greece to compare renal prognosis between a group that started ESA treatment early and a group that deferred ESA treatment. They found that renal prognosis in the early ESA treatment group was significantly better [8], which is similar to the findings in the present study.

This study supports the recommendation in the 2008 guideline for renal anemia in CKD patients presented by the Japanese Society for Dialysis Therapy, which states that ESA therapy should be started when the Hb level is less than 11 g/dL in non-dialysis patients [14]. It is necessary to consider each patient’s condition when deciding the appropriate time to start treatment, as recommended in the 2013 Evidence-based Clinical Practice Guideline for CKD published by the Japanese Society of Nephrology [15].

This study has some limitations. Because this is an observational study, no randomization was used in comparison of Japanese patients based on the Hb levels at initiation of epoetin beta. The timing of epoetin beta initiation depended on each nephrologist evaluating the patient’s total physical condition, and subjective bias might have affected the results of this study. Thus, the findings should be interpreted with this in mind, although the IPW method was used to adjust for artificial censoring before calculating hazard ratios for the primary endpoint. To account for lead-time bias, the date, Hb level, and sCr level at the time Hb levels decreased below 11 g/dL were retrospectively collected, but data on albumin and urinary protein were not. Therefore, analysis of renal events using the IPW method could not be adjusted for albumin or urinary protein values. Also, we were unable to capture patients with events occurring before treatment initiation, which could bias results in favor of the deferred treatment initiation group. Response and resistance to ESAs were also not evaluated in this study, and missing data might have affected the results.

## Conclusion

Initiation of ESA therapy when Hb levels decreased below 11 g/dL but not below 10 g/dL could be more effective at reducing the risk of renal events in non-dialysis CKD patients with anemia compared with initiation of ESA therapy at below 9 g/dL or even 10 g/dL.
